# Misdiagnosis and Coinfection of Localized Pulmonary Histoplasmosis with Pulmonary Tuberculosis: A Systematic Review of Published Cases

**DOI:** 10.3390/jof12030190

**Published:** 2026-03-06

**Authors:** Sem Samuel Surja, Donnatella Valentina, Anita Devi Krishnan Thantry, Jonathan Christianto Subagya, Edho Yuwono, Darmadi Darmadi, Nisa Fauziah, Robiatul Adawiyah, Retno Wahyuningsih

**Affiliations:** 1 Doctoral Program in Biomedical Sciences, Faculty of Medicine, Universitas Indonesia, Jakarta 10430, Indonesia; 2 Department of Parasitology, School of Medicine and Health Sciences, Atma Jaya Catholic University of Indonesia, Jakarta 14440, Indonesia; sem.samuel@atmajaya.ac.id (S.S.S.); donnatellavalentina333@gmail.com (D.V.); subagyajonathan@gmail.com (J.C.S.); 3 Faculty of Medicine, Manipal University College Malaysia, Melaka 75150, Malaysia; anita.krishnan@manipal.edu.my; 4 Department of Parasitology, Faculty of Medicine, Universitas Kristen Indonesia, Jakarta 13630, Indonesia; edho.yuwono@uki.ac.id; 5 Department of Internal Medicine, Faculty of Medicine, Universitas Sumatera Utara, Medan 20155, Indonesia; darmadi@usu.ac.id; 6 Division of Parasitology, Department of Basic Biomedical Science, Faculty of Medicine, Universitas Padjadjaran, Bandung 40161, Indonesia; nisa@unpad.ac.id; 7 Research Center for Care and Control of Infectious Disease, Universitas Padjadjaran, Bandung 40161, Indonesia; 8 Department of Parasitology, Faculty of Medicine, Universitas Indonesia, Jakarta 10430, Indonesia; robiatul.adawiyah01@ui.ac.id; 9 Parasitology Laboratory, Universitas Indonesia Hospital, Depok 16424, Indonesia; 10 Infectious Disease and Immunology Research Centre (IMERI), Faculty of Medicine, Universitas Indonesia, Jakarta 10430, Indonesia; 11 Dharmais Cancer Hospital, Jakarta 11420, Indonesia

**Keywords:** histoplasma, pulmonary tuberculosis, coinfection, diagnostic errors, X-rays

## Abstract

Pulmonary histoplasmosis is often misdiagnosed as or coinfected with pulmonary tuberculosis (TB). This study aims to analyze the misdiagnosis or co-occurrence of published cases of pulmonary TB and pulmonary histoplasmosis. Cases of histoplasmosis with dissemination were excluded, as it affects other organs. Systematic research was conducted using PubMed, EBSCOhost, ProQuest, BioRxiv, and MedRxiv databases. Twenty-seven articles were included, covering a total of 51 cases. Males were predominantly affected, with a median age of 54 years. Exposure to caves and farming occupations were identified as the primary sources of infection (61.9%). The most common clinical symptoms were fever (80%) and cough (82.5%). Laboratory tests revealed culture positivity in 77.1% of cases, with sputum being the most frequently used specimens. In proven pulmonary histoplasmosis, antibody tests were positive in 18 out of 24 cases. Chest X-rays commonly showed cavities, infiltrates, and nodules, with an increase in nodular pattern in recent cases. The number of pulmonary nodules detected was higher on chest computed tomography (CT). Radiologic abnormality could occur in any lung region. This review suggests the potential for misdiagnosis and/or coinfection of pulmonary histoplasmosis and pulmonary TB. The combination of clinical suspicion, radiological findings, antibody and/or antigen testing could improve the diagnosis of pulmonary histoplasmosis.

## 1. Introduction

Histoplasmosis caused by the fungus *Histoplasma capsulatum* is highly endemic in regions of North, Central, and South America, and it is also reported in certain countries of Asia and Africa [1]. The highest endemicity in North America is found in the valleys of the Mississippi and Ohio Rivers in the central and eastern United States, with an estimated incidence of 6.1 cases per 100.000 population. Approximately 80–90% of the population in these areas will be exposed to *Histoplasma* during their lifespan. In Central and South America, the prevalence of infection may exceed 30%. Seroprevalence rates vary widely across regions, ranging from 0.1% in Chile, 20% in Peru, 35–40% in Argentina, and nearly 90% in certain areas of Brazil [2].

Histoplasmosis can manifest in several forms. The acute pulmonary histoplasmosis typically manifests as a subclinical or self-limited respiratory illness. However, in immunocompromised patients or following the inhalation of a large inoculum, a flu-like illness may occur, characterized by fever, chills, malaise, headache, weakness, shortness of breath, dry cough, chest discomfort, and pleuritic chest pain. These symptoms usually improve within one month in most patients [2,3]. Chronic pulmonary histoplasmosis (CPH) usually occurs in middle-aged patients, with a male predominance, and it is often associated with underlying structural lung disease and a history of smoking. Respiratory symptoms include a productive cough, dyspnea, pleuritic chest pain, and hemoptysis, in addition to constitutional symptoms such as fever, chills, night sweats, anorexia, and weight loss. The duration of symptoms can last from months to years. Persistent cavitation, pulmonary fibrosis, pleural thickening, and pulmonary nodules may develop [1,2,3]. Disseminated histoplasmosis is mostly reported in immunocompromised people living with HIV (PLHIV). The symptoms of disseminated histoplasmosis are nonspecific and may be indistinguishable from those of other infectious diseases [4]. Common symptoms include fever, fatigue, night sweat, weight loss, hepatosplenomegaly, and respiratory symptoms [3,5].

Histoplasmosis is frequently misdiagnosed as or coexists with pulmonary tuberculosis (TB). The predisposition of pulmonary histoplasmosis to cause cavitation in the apical and apico-posterior segments of the upper lung lobes is similar to that of pulmonary TB and chronic pulmonary aspergillosis (CPA) [1]. Pulmonary histoplasmosis may be misdiagnosed as pulmonary TB, especially when sputum smear, culture, or GeneXpert tests are negative [2,6].

In countries with high TB burden such as India, Indonesia, China, Nigeria, and South Africa [7], pulmonary TB and pulmonary histoplasmosis may also occur concurrently in the same patient. Ekeng et al. (2022) conducted a study in Nigeria and reported that positive *Histoplasma* antigen or PCR was found in 7.4% of patients with positive TB PCR results, indicating probable TB–histoplasmosis co-occurrence, while 16.8% positivity among patients with negative TB PCR results suggested misdiagnosis [8]. Wijaya et al. (2024) reported that 11.5% of patient sera with TB were positive for *Histoplasma* galactomannan antigen testing, supporting possible pulmonary histoplasmosis coinfection among TB patients [9].

Recent studies in Indonesia have also reported positive *Histoplasma* antibody results in pulmonary TB patients. Dewi et al. (2023) found that 16.9% of patients with bacteriologically confirmed TB and 4% of patients with clinical TB (not bacteriologically confirmed) had antibodies to *H. capsulatum* [10]. A nationwide study by Kusmiati et al. (2023) found that 12.7% of pulmonary TB patients (39 out of 306 patient sera) had antibodies to *H. capsulatum* [11]. Another study by Soeroso et al. (2024) reported antibodies to *H. capsulatum* in one third of patients’ multidrug resistance (MDR) TB [12]. Although antibody testing could not differentiate between active disease and past exposure, these findings, accompanied by a suggestive clinical presentation, raise suspicion for CPH in a TB-endemic setting. Other factors such as low clinical suspicion of histoplasmosis, difficulty distinguishing histoplasmosis from other infectious tropical diseases, lack of diagnostic facilities, and lack of health care funding may contribute to under-detection of histoplasmosis, resulting in both misdiagnosis and unrecognized TB–histoplasmosis co-occurrence [1,11].

Studies assessing the clinicoradiological profile of patients with pulmonary histoplasmosis that is initially misdiagnosed as or coinfected with pulmonary TB remain scarce. While pulmonary histoplasmosis is the most commonly found clinical spectrum of histoplasmosis, most research focused on disseminated histoplasmosis, rather than pulmonary histoplasmosis [2]. Therefore, a systematic review examining case reports and articles that focus on misdiagnosed pulmonary histoplasmosis, as well as the coinfection of both pulmonary TB and pulmonary histoplasmosis, will provide insight into diagnostic parameters and the management of such cases. The aim of this review is to analyze published cases of localized pulmonary histoplasmosis misdiagnosis as or co-occurrence with pulmonary TB.

## 2. Materials and Methods

### 2.1. Study Design and Setting

This systematic review was conducted in accordance with the Preferred Reporting Items for Systematic Reviews and Meta-analysis (PRISMA) statement guidelines to identify pulmonary histoplasmosis misdiagnosed as or coinfected with pulmonary TB. The literature search was performed using the following five databases: PubMed, EBSCOhost, ProQuest, MedRxiv, and BioRxiv. The general search terms used include ‘histoplasmosis’, ‘tuberculosis’, ‘pulmonary’, ‘misdiagnosis’, and ‘coinfection’. The complete keywords are listed in Table 1. No limitations of the publishing period were imposed.

### 2.2. Study Eligibility Criteria

Inclusion criteria for studies to be included should meet the following criteria based on the PCC framework. The population (P) consisted of subjects diagnosed with pulmonary histoplasmosis, particularly those who were misdiagnosed as or coinfected with pulmonary TB. The condition (C) referred to pulmonary histoplasmosis, with a focus on demographic characteristics, clinical manifestation, imaging findings (chest X-ray (CXR) and chest computed tomography (CT)), and diagnosis method (culture, histopathology, sputum smear, PCR, and antigen and antibody testing). The context (C) included studies conducted in any geographic region that mentioned a correlation between pulmonary histoplasmosis and pulmonary TB. The criteria for studies to be excluded were the following: (1) any form of reviews, including systematic review and meta-analysis, except those which included a brief report of a case in the paper; (2) not using English language; (3) only mentions cases of disseminated histoplasmosis or disseminated TB; (4) cases of pulmonary histoplasmosis but with dissemination to other organs; (5) articles that did not mention the clinicoradiological profile of individual patients. The protocol of this systematic review was registered with the open science framework (OSF) registries and can be accessed through the link https://doi.org/10.17605/OSF.IO/ES9YH.

### 2.3. Data Screening and Selection

Database searching was conducted independently by two reviewers (DV and JS) using keywords, and duplicate titles were removed using the Mendeley application. Titles and abstracts were screened separately. Full texts of the included studies were assessed independently. In case of discrepancies, further discussion was held with SS, AT, and RW. Two reviewers (SS and EY) independently assessed the quality of each included article. We used the Joanna Briggs Institute (JBI)’s Critical Appraisal Checklist for Case Reports, which consists of eight items (https://jbi.global/, accessed on 29 January 2025) with four response options as follows: yes, no, unclear, and not applicable. Data extraction and synthesis were conducted by DV and JS, with final validation by SS and RW.

### 2.4. Data Extraction

The following data were extracted from each eligible study: (1) the authors and year of publication; (2) demographic and baseline characteristics of patients including their sex, age, country of residence, occupation or presumed source of exposure, and comorbidities; (3) clinical manifestations; (4) imaging results from CXR and chest CT; and (5) laboratory results and investigation methods done to diagnose histoplasmosis.

### 2.5. Data Synthesis

All data were presented in table format and expressed as numbers, percentages, or simple statistical calculations, such as the median. Age was classified using interquartile range. Cases were also classified as either misdiagnosis or coinfection. Misdiagnosis of TB was defined as an incorrect diagnosis of histoplasmosis as TB. This included cases where anti-tuberculosis drugs were administered despite negative results from acid-fast bacilli (AFB) testing, TB culture, and TB PCR. In this study, considering TB as a differential diagnosis, evidenced by clinical findings similar to TB and conducting TB tests such as the tuberculin test, AFB testing, TB culture, and TB PCR, with negative results, was also classified as misdiagnosis. Coinfection was defined as the simultaneous presence of both histoplasmosis and TB in a single patient. A TB diagnosis requires microbiological confirmation through AFB testing, TB culture, or TB PCR. Imaging results were quantified based on all cases. Considering the increasing utilization of chest CT for TB in 1998 among the cases included in this systematic review, imaging results were further quantified for cases reported before and after 1998.

Additional analyses were performed for laboratory and investigation methods, based on case definition of histoplasmosis. In this study, pulmonary histoplasmosis was categorized as proven, probable, or possible based on the following criteria. Proven pulmonary histoplasmosis was determined by both of the following: (1) positive symptoms of pulmonary histoplasmosis, AND (2) positive histopathology test OR positive culture OR positive molecular-based testing from respiratory samples (e.g., sputum, BAL, or pulmonary tissue culture). Probable pulmonary histoplasmosis was determined in patients meeting both of the following: (1) positive symptoms of pulmonary histoplasmosis (duration not specified), AND (2) positive antigen testing. Possible pulmonary histoplasmosis was determined by both of the following: (1) positive symptoms of pulmonary histoplasmosis, AND (2) positive antibody testing. The number of antigen and antibody tests that were positive in proven pulmonary histoplasmosis cases was also recorded.

## 3. Results

**Figure 1:**
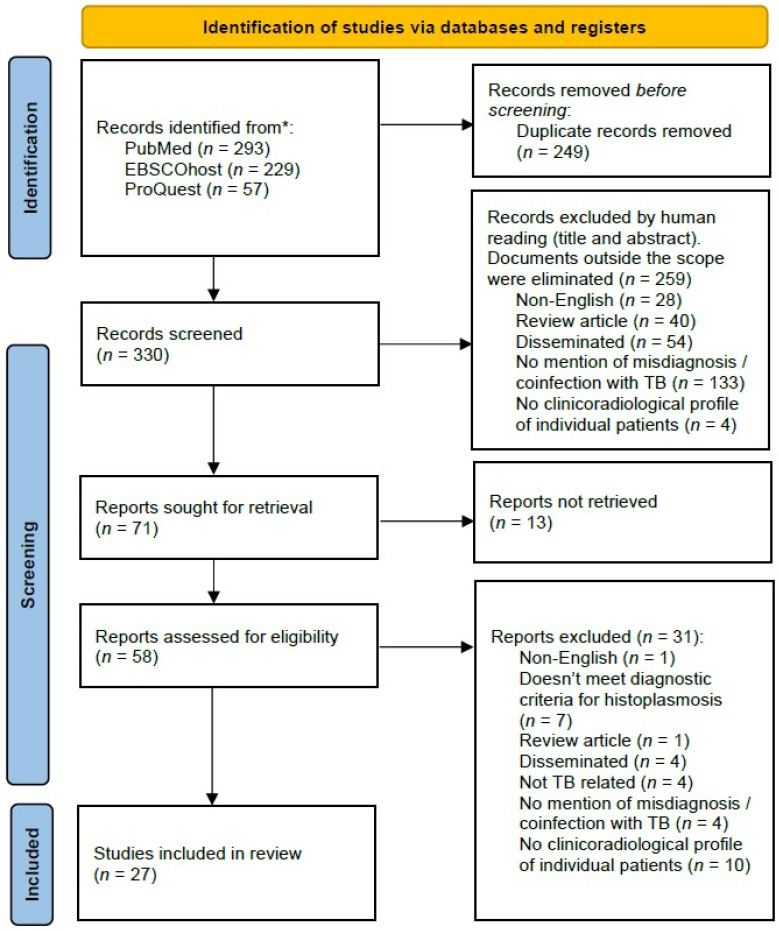
PRISMA schematic diagram for the screening and selection of eligible studies. A detailed list of studies excluded at the full-text review stage, along with the reasons for exclusion, is presented in Supplementary Table S1. The completed PRISMA 2020 checklist is available in Supplementary Table S2. * Records in a language other than English (German, Spanish, French, Turkish, Japanese, Russian, or Chinese) were excluded. Full papers are not available.

A total of 579 studies were identified across three databases. After removing duplicates and screening the records, 27 studies were included in this systematic review, covering the period from 1949 to 2018 [13,14,15,16,17,18,19,20,21,22,23,24,25,26,27,28,29,30,31,32,33,34,35,36]. From these 27 studies, there was no duplicate or overlapping patient case and a total of 51 cases were reported and reviewed (Figure 1).

The quality of the included studies was assessed using the JBI quality appraisal, as presented in Table 2. All 27 articles met the majority of the quality indicators, although there was some variability in specific items. Some cases were described in detail, covering demographic characteristics, risk factors, patient history, clinical manifestations, examinations performed, and treatments given. Others provided only a brief description of certain checklist points but still contributed valuable data for review in this article. There were also review articles that included relevant patient cases (Hage et al., 2015 and Gurney et al., 1996 [20,21]), making them suitable for inclusion in this systematic review.

### 3.1. Demographic and Baseline Characteristics

**Figure 2:**
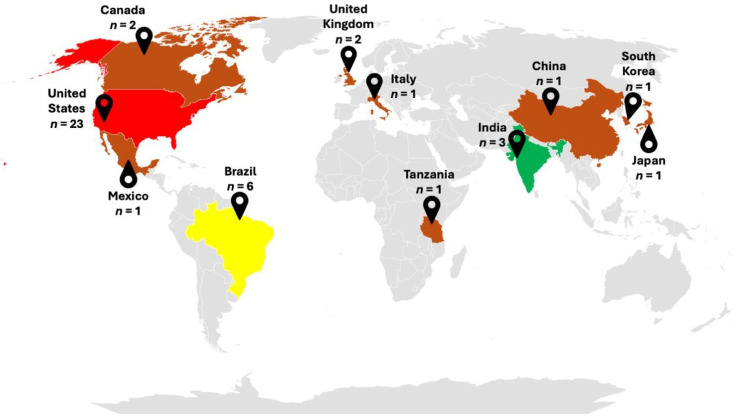
Mapping of recorded misdiagnosis or coinfection of pulmonary histoplasmosis with pulmonary TB. Red, yellow, and green colors represent countries with the highest, second highest, and third highest number of cases, respectively. Brown color signifies countries with a small number of cases.

Among 51 recorded cases of TB-related histoplasmosis, geographical data were available for 44 of them. The majority of cases originated from the United States and Brazil, which accounted for 66% of the cases (Figure 2). The study found that males were affected four to five times more frequently than females, with a median age of 54 years old. Data regarding the source of exposure were available for only 21 cases [14,16,18,19,22,29,30,31,32,33,36,40]. Among them, travel (mostly to caves) and agricultural occupation were the most common, accounting for 61.9% sources of exposure. Other risk factors included occupations involving contact with caves, soil, wood, rock, and water. Most cases were pulmonary histoplasmosis misdiagnosed as TB (76.5% of the cases; Table 3). No clear pattern of comorbidities was identified; however, some patients were reported to have conditions requiring steroid treatment, such as rheumatoid arthritis (*n* = 2 [27,35]) and kidney transplantation (*n* = 2 [26,38]); none of the patients were HIV-positive.

### 3.2. Clinical Manifestation

Clinical examination upon admission revealed fever in 80% of patients, with a median duration of 14 days (range: 7–365 days). Body temperature varied across the cohort. Cough was present in 82.5% of patients. Among the 14 cases with duration data, 57.1% (*n* = 8) reported a cough lasting more than 8 weeks. The median cough duration was 45 days and productive cough was more prevalent (61.9%, Table 4).

Other symptoms were nonspecific, including dyspnea (47.5%), weight loss (35%), sweat/night sweat (35.1%), and fatigue/malaise/weakness/asthenia/adynamic (37.5%), with a median duration exceeding 4 weeks. Hemoptysis was also observed in 12.5% of patients (Table 4). Laboratory data showed that anemia was present in six out of eight patients, while leukocytosis was present in three out of nine patients. Neutrophil counts were generally within the normal range, though most were near the upper limit. Only one patient exhibited a high neutrophil count. Elevated ESR occurred in all patients.

### 3.3. Imaging Findings

CXRs were mentioned in 39 patients. On CXR, cavity and infiltrate were found in 19 cases (48.7% patients) and 14 cases (35.9% patients), respectively. Pulmonary nodule was only found in 25.6% of all cases. A comparison of imaging presentation pre- and post-1998 was also conducted. Interestingly, nodule (50%) was the most commonly found CXR feature after 1998 (Table 5). Cavity was also found, but in only 22.2% of the patients. As for chest CT, it was only performed in 11 cases. Pulmonary nodules were found in nine cases (81.8%), in which three out of nine pulmonary nodules were not visible on CXR. Nodule sizes were variable; however, no mass or >30 mm lesion size was recorded. Cavity is the second most common feature of pulmonary CT. The other features were variable and similar with TB.

Radiologic abnormalities in the cases were predominantly located in the upper lung lobes. When cases before and after 1998 were compared, an interesting trend emerged. Prior to 1998, the majority of abnormalities were confined to the upper lobes (18 out of 21 cases). However, after 1998, the distribution of lesions became more diverse. Among the twenty-two cases analyzed, four involved the upper lobe, while four others presented with extensive lesions affecting the upper lobe. Notably, two cases involved the middle lobe, five cases were localized in the lower lobe, and five cases exhibited a miliary pattern.

### 3.4. Laboratory Findings

Laboratory findings were mentioned in 39 cases. Culture was positive in 77.1% of 35 cases (Table 6). The clinical specimens used were mostly sputum, lung tissue, and BAL (Table 7). Various culture mediums were employed, including Sabouraud dextrose agar (SDA); plain brain heart infusion (BHI) agar; BHI agar with the addition of 10% whole blood, 40 units of streptomycin, and 20 units of penicillin per cubic cm of culture medium; and mycobiotic agar. No specific culture techniques were reported.

Antibody and antigen testing were performed in twenty-seven and three patients, respectively (Table 6). Antibody was positive in 21 cases (77.8% of 27 cases, Table 6). Three patients exhibited only antibody positivity, without any other positive laboratory testing result. In these cases, suggestive radiological findings and successful empirical treatment confirmed the diagnosis. Antigen testing was positive only in one case (Table 6). Of those with proven pulmonary histoplasmosis, antibody testing was conducted on 24 patients, of which 18 were positive (Table 8).

## 4. Discussion

Histoplasmosis was first described in 1906 by American pathologist Samuel T. Darling. He discovered the condition during an autopsy of a person in Panama who was initially suspected to have miliary TB due to the similarity in presentation. Upon further investigation, Darling found capsule-like cells within the body tissues, particularly in the lungs, liver, spleen, bone marrow, and other organs. These cells were later named *H. capsulatum* [41]. Since then, the misdiagnosis of histoplasmosis as TB or coinfection has been occurring [1]. This review article included all recorded cases of pulmonary histoplasmosis that were either misdiagnosed as pulmonary TB or coinfected with pulmonary TB. Unfortunately, meta-analysis could not be conducted due to the variability of the cases.

Our review indicates that pulmonary histoplasmosis cases were reported most frequently in the United States and Brazil, which is consistent with previous reports [2,42]. The risk factors identified include male gender, old age, and the occupations involving exposure to bird or bat droppings indoors or dust from environmental disruption, similar to previous knowledge [2]. A study conducted in Indonesia also found that positive antibody testing was predominantly found in TB patients who lived in damp houses, had a history of smoking, and worked in agriculture, which may also be a source of infection [43]. This study confirms that a patient’s country or region of origin and occupation can provide important clues about the source of infection, which is crucial for clinical suspicion and early diagnosis [44]. Also, in this study, no specific comorbidity was found. All PLHIV were excluded due to signs of dissemination to other organs, which is consistent with previous data that over 95% of the patients with AIDS will present with progressive disseminated disease [2,45]. Interestingly, no CPH cases were reported from Southeast Asia, despite the high positivity of histoplasmin skin tests and the prevalence of TB in certain areas of countries like Indonesia, Myanmar, and Thailand [46,47].

Fever and cough are among the most found symptoms. The duration of both symptoms is varied and could last weeks before diagnosis, which suggests possible chronicity of the pulmonary histoplasmosis. Fever and cough were also the main clinical symptoms found in Bourne-Watrin et al. (2023), and they occurred in 89% and 58% patients with pulmonary histoplasmosis coinfected with HIV, respectively [48]. Other reports of CPH found that cough was the most common symptom (85%), with fever occurring in 76% of patients [49]. Modern case series in United States 2018–2019, which include the full histoplasmosis spectrum, also report cough and fever in more than 60% of patients [44]. It is concluded that common pulmonary histoplasmosis and TB-related histoplasmosis share similar main symptoms.

Productive coughs were more common (61.9%) among patients in this review. This number is notably higher than the typical cases of CPH. Goodwin et al. (1976) found that 42% of patients with CPH in a TB sanatorium in the United States had productive sputum, while only 14% of PLHIV with pulmonary histoplasmosis had productive sputum [50]. This discrepancy could pose the risk of bias since productive cough permits easier culture and diagnosis. Bronchoscopy with a BAL sample is the best method for diagnosing pulmonary histoplasmosis, especially in cases with nonproductive cough [2]. Therefore, it is suspected that the symptomatic cases with nonproductive cough might be missed in resource-limited settings, particularly in Asian countries with less access to modern diagnostic testing [51].

In addition to the common symptoms, other manifestations such as dyspnea, weight loss, sweating, fatigue/malaise/weakness/asthenia/adynamic, and hemoptysis could also be found. All these symptoms were chronic and nonspecific. All the symptoms were like those observed in pulmonary TB, as also noted in another review [46]. The laboratory data in this study were inconclusive, because the hematologic changes are not as clear as in disseminated histoplasmosis [52]. Only ESR showed high level in all patients tested, indicating inflammations [53].

Cavities, infiltrates, and nodules were the most frequently reported radiological findings in this review, with an apparent increase in nodular patterns observed in more recent cases. We also noted an interesting finding in which three of the nine pulmonary nodules detected on chest CT were not visible on CXR. The observation aligns with that of Kennedy et al. (2007), who found that, in patients with CPH, only 30% had cavities while 93% exhibited nodule in chest CT [49]. In the same study, nodules and cavities were found in 74% and 28% patients by CXR, respectively. Bourne-Watrin et al. (2023) [48] reported only 25% nodule in the CXR, while more nodule (83%) was found in chest CT and mostly in the form of micronodules (<3 mm). This discrepancy suggests that smaller nodules in certain locations may be missed on CXR [54,55]. These observations in pulmonary histoplasmosis misdiagnosed or coinfected with pulmonary TB warrant confirmation in larger, systematically designed studies.

Most cases in this study exhibited radiologic abnormalities in the upper lung field, although the involvement of the middle and lower lung fields was observed in a few cases after 1998. Singh et al. (2015) reported that only 10% of pulmonary TB cases involved the lower lung field, and most of the cases had comorbidities such as diabetes, HIV infection, renal disease, and corticosteroid therapy [56]. Histoplasmosis, however, can affect all lung fields [57]. Denning et al. (2025) [58] suggested that chronic cavitary pulmonary histoplasmosis mostly affected the upper lobe, although in some cases it could occur in other lobes. In contrast, pulmonary nodules typically have lower lobe predominance.

CXR is one of the most common tests conducted for TB in resources-limited countries. However, in several countries, rapid molecular testing like GeneXpert is used as the main diagnostic tool for TB [59]. Therefore, key findings for pulmonary histoplasmosis may be overlooked. The current review includes older cases of TB-related pulmonary histoplasmosis. The results found in this study possibly depict the situation in a resource-limited TB endemic country which used CXR rather than chest CT. A recent study by Wijaya et al. (2024) found 11.5% of patients with clinical TB, as suggested by typical symptoms and positive radiological findings without bacteriological evidence, were positive for *Histoplasma* galactomannan antigen [9]. It is suggested that one should be cautious of pulmonary histoplasmosis or other fungal diseases, especially when GeneXpert TB is negative.

Histoplasmosis was mostly diagnosed through culture. Culturing *H. capsulatum* in clinical samples is challenging. Due to its slow growing nature, *H. capsulatum* could be overgrown by other respiratory pathogens or saprophytes [60]. The utilization of a more selective culture medium is needed if histoplasmosis is suspected. An example of this is SDA supplemented with chloramphenicol and cycloheximide to prevent bacterial and fungal overgrowth, especially *Candida* which saprophytes in the upper respiratory tract [9,13,61]. Additionally, although more invasive techniques could produce a higher yield, a combination with other techniques, such as histopathology, could increase sensitivity [48]. The presentation of yeast cells in sputum microscopy must be interpreted with caution since *Candida* and other yeast can be found in the upper respiratory tract.

This study included primarily cases of immunocompetent patients with chronic pulmonary symptoms suggestive of pulmonary histoplasmosis and analyzed the diagnostic performance of antibody and antigen testing for its diagnosis. Antibody testing was positive in 18 of 24 proven pulmonary histoplasmosis cases. Five cases underwent immunodiffusion testing with the detection of both H and M bands, which is considered confirmatory for histoplasmosis, while the other 16 cases were tested using complement fixation, a non-confirmatory method that supports the diagnosis of probable histoplasmosis [62]. In all cases categorized as proven histoplasmosis, positive antibody results were accompanied by positive culture or histopathology findings. However, due to the limited sample size and variability in antibody testing methods, these results should be interpreted with caution and validated in larger studies.

Due to the chronic nature of pulmonary histoplasmosis, antibody testing is particularly useful in diagnosis, especially in patients living in an endemic area with strongly suggestive clinical features. In the absence of positive culture or histopathology, the diagnosis of CPH may be supported by paired-serum antibody testing or immunodiffusion with H-band detection, in combination with compatible clinical features and regional endemicity [62]. This finding is consistent with those of Hage et al. (2019), who also recommended the use of serological testing in immunocompetent patients with suspected pulmonary histoplasmosis [63]. Several reports from Indonesia, a TB-endemic country, found a high number of positive antibody testing for histoplasmosis in bacteriologically confirmed and clinical pulmonary TB patients [10,12]. Clinicians should be aware of the possibility of misdiagnosis or coinfection with CPH, especially in cases where the laboratory diagnosis of TB is negative and TB therapy fails to achieve clinical improvement [9]. Because only three proven histoplasmosis cases included antigen testing in the reports, a clear conclusion could not be made. Further study assessing the usage of antigen testing, especially in pulmonary histoplasmosis among TB patients in many parts of the world, should be conducted.

This is the first study that reviewed the clinicoradiological profile of published cases of pulmonary histoplasmosis in TB patients. Pulmonary histoplasmosis is often misdiagnosed as or coinfected with pulmonary TB, due to the similarities of the clinical symptoms. This review provides insight into the common clinical symptoms, radiological findings, and diagnostic methods that could aid in the suspicion of pulmonary histoplasmosis among pulmonary TB patients. This study is particularly important given that pulmonary histoplasmosis is the most common clinical presentation in histoplasmosis and that, in TB-endemic countries, it could be confused with TB.

This study has several limitations. First, the literature search was restricted to English language article, which represents a significant limitation. This may have led to the exclusion of relevant studies from highly endemic regions, particularly in Asia and Latin America, where valuable information might be published in the local languages. Second, this review deliberately focused on isolated pulmonary histoplasmosis and excluded disseminated disease, although disseminated histoplasmosis may also involve the lungs. This restriction was applied to minimize clinical heterogeneity due to multi-organ involvement and diverse underlying conditions. Therefore, only a small number of cases were included, and no PLHIV were identified. Future studies focusing on disseminated histoplasmosis with pulmonary involvement should be conducted separately, as the clinic-radiologic profiles are likely distinct from those of isolated pulmonary histoplasmosis and classical pulmonary TB.

## 5. Conclusions

This systematic review highlights the potential for misdiagnosis or coinfection of pulmonary histoplasmosis and pulmonary TB. Pulmonary histoplasmosis cases are likely underdiagnosed due to the many similarities with TB, including overlapping clinical manifestations and imaging findings. Living in a histoplasmosis-endemic area with a high burden of TB, being male and middle-aged, presenting with fever and cough, and demonstrating radiological findings such as cavities, infiltrate, or pulmonary nodule that can appear in any lung region are important clues for pulmonary histoplasmosis in a TB patient. While CXR could be useful in early suspicion, chest CT could provide a clearer picture. Antibody detection could aid in the diagnosis, although access may be difficult in many countries. We recommend performing further assessment with fungal culture, fungal serology, and a combination of fungal serology and antigen testing in culture-negative TB or atypical TB presentation.

This review likely reflects the situation in developing countries that are still grappling with TB cases and have limited testing for histoplasmosis. Further studies are needed to investigate histoplasmosis in Asian countries, especially those with a high positivity rate for the histoplasmin test and high endemicity of TB. A combination of clinical suspicion, radiological findings, and antibody and/or antigen testing could improve the diagnosis of pulmonary histoplasmosis.

## Figures and Tables

**Table 1:** Search strategy and number of records.

Search Combination	Records
PubMed	
(((((“*histoplasma*”[MeSH Terms]) OR (“histoplasmosis”[MeSH Terms])) OR (“histoplasmosis”[Title/Abstract])) OR (“*histoplasma*”[Title/Abstract])) AND ((“tuberculosis”[Title/Abstract]) OR (“tuberculosis”[MeSH Terms]))) AND (((“pulmonary”[Title/Abstract])) OR (“respiratory”[Title/Abstract]))	293
EBSCOhost	
(AB *histoplasma* OR AB histoplasmosis) AND (AB tuberculosis OR AB (tuberculosis or tb)) AND (AB respiratory OR AB pulmonary)	229
ProQuest	
(*histoplasma* OR Histoplasmosis OR histoplasmosis) AND (tuberculosis OR Tuberculosis OR (about tuberculosis) OR (active tuberculosis)) AND pulmonary OR respiratory	57
BIORXIV	
(*histoplasma* OR Histoplasmosis OR histoplasmosis) AND (tuberculosis OR Tuberculosis) AND (respiratory OR Respiratory)	0
MEDRXIV	
(*histoplasma* OR Histoplasmosis OR histoplasmosis) AND (tuberculosis OR Tuberculosis) AND (respiratory OR Respiratory)	0

**Table 2:** Joanna Briggs Institute (JBI)’s appraisal criteria to evaluate the studies.

Author, Year	JBI Appraisal Items and Score								Overall Appraisal
	1	2	3	4	5	6	7	8	
Capone et al., 1999 [13]	√	√	√	√	√	√	X	√	Included
Chen et al., 2024 [37]	√	√	√	√	√	√	X	√	Included
Cottle et al., 2013 [14]	√	√	√	√	√	√	X	√	Included
Dutta et al., 2018 [15]	√	√	√	√	X	X	X	√	Included
Gandhi et al., 2015 [16]	√	√	√	√	√	√	X	√	Included
Gascon et al., 2000 [17]	-	√	√	√	√	√	X	√	Included
Goodwin et al., 1965 [18]	√	√	√	√	√	√	X	√	Included
Goodwin et al., 1967 [19]	√	√	√	√	√	√	X	√	Included
Gurney et al., 1996 [20]	√	X	√	√	X	X	X	√	Included *
Hage et al., 2015 [21]	X	√	√	√	√	√	X	√	Included *
Kabangila et al., 2011 [22]	√	√	√	√	√	√	X	√	Included
Kajfasz et al., 2012 [23]	√	√	√	√	√	√	X	√	Included
Kandi et al., 2016 [24]	√	√	√	√	√	X	X	√	Included
Lee et al., 2018 [25]	√	√	√	√	√	√	X	√	Included
Lobo et al., 2014 [26]	√	√	√	√	√	√	X	√	Included
Lum et al., 2018 [27]	√	√	√	√	√	√	X	√	Included
Monroe et al., 1952 [28]	√	√	√	√	√	√	√	√	Included
Oliveira et al., 2005 [29]	√	√	√	√	√	√	X	√	Included
Pometta et al., 1999 [30]	√	√	√	√	√	√	√	√	Included
Post et al., 1957 [31]	√	√	√	√	√	√	X	√	Included
Pugsley et al., 1963 [32]	√	√	√	√	√	√	X	√	Included
Saliba et al., 1962 [33]	√	√	√	√	√	√	√	√	Included
Salzman et al., 1988 [34]	√	√	√	√	√	√	X	√	Included
dos Santos et al., 2009 [35]	√	√	√	√	√	√	X	√	Included
Shimamoto et al., 2007 [38]	√	X	√	√	√	√	√	√	Included
Tustin et al., 1980 [39]	√	√	√	√	√	√	√	√	Included
Wilson et al., 1949 [36]	√	√	√	√	√	√	X	√	Included

* Review article containing a brief case report that was still relevant to be included. (√): Yes; (X): no; (-): unclear. Item 1: Were the patient’s demographic characteristics clearly described? Item 2: Was the patient’s history clearly described and presented as a timeline? Item 3: Was the current clinical condition of the patient on presentation clearly described? Item 4: Were the diagnostic tests or methods and the results clearly described? Item 5: Was the intervention(s) or treatment procedure(s) clearly described? Item 6: Was the post-intervention clinical condition clearly described? Item 7: Were adverse events (harms) or unanticipated events identified and described? Item 8: Does the case report provide takeaway lessons?

**Table 3:** Demographic and baseline characteristics.

Characteristics	No/Total (%) Median [Range]	References
Sex ratio M:F	4.5:1	[13,14,15,16,18,19,20,22,24,25,26,27,28,29,30,31,32,33,35,36,37,38,39]
Median age (years) (*n* = 44)	54 [15–77]	
15–35 years old	7/44 (15.9)	[14,16,19,22,33,37]
36–56 years old	18/44 (40.9)	[16,18,19,26,27,28,29,30,33,36,38,39]
57–77 years old	19/44 (43.2)	[13,15,16,18,19,20,24,25,28,29,31,32,35]
Country of residence (*n* = 44)		
USA	23/44 (52.3)	[18,19,20,27,28,31,34,36]
Brazil	6/44 (13.6)	[13,29,35]
Mexico	3/44 (6.8)	[16]
India	3/44 (6.8)	[15,24,26,32]
Canada	2/44 (4.5)	[32]
UK	2/44 (4.5)	[14]
Tanzania	1/44 (2.3)	[22]
Korea	1/44 (2.3)	[25]
Italy	1/44 (2.3)	[30]
China	1/44 (2.3)	[37]
Japan	1/44 (2.3)	[38]
Source of exposure/occupation (*n* = 21)		
Travel	4/21 (19)	[14,16,30,40]
Caves	2/4 (50)	[14,18,19,32,33,40]
Farm	9/21 (42.9)	[18,19,32,33,38]
Craftsmanship (working with stones and metals)	3/21 (14.3)	[22,36]
Attic with bat guano	2/21 (9.5)	[29]
Construction	2/21 (9.5)	[16,19]
Laundry work	1/21 (4.8)	[33]
Coinfection/misdiagnosis		
Coinfection	12/51 (23.5)	[13,15,19,22,33,34]
Misdiagnosis	39/51 (76.5)	[13,14,16,17,18,19,20,21,23,24,25,26,27,28,29,30,31,32,33,35,36,37,38,39]

**Table 4:** Clinical symptoms.

Clinical Symptoms	No/Total (%) Median (Range)
Fever	32/40 (80)
Median temperature (°C) (*n* = 7)	38.8 (37.8–40.3)
Median duration of fever (days) (*n* = 11)	14 (7–365)
Cough	33/40 (82.5)
Productivity (*n* = 21)	
Productive	13/21 (61.9)
Nonproductive	8/21 (38.1)
Chronic cough * (*n* = 14)	8/14 (57.1)
Median duration of cough (days) (*n* = 14)	45 (5–1440)
Dyspnea	19/40 (47.5)
Median duration of dyspnea (days) (*n* = 5)	30 (5–1440)
Weight loss	14/40 (35)
Median weight lost (kilograms) (*n* = 6)	8.5 (3.2–11.8)
Median duration of weight loss (days) (*n* = 8)	150 (8–1440)
Sweats/Night sweats	13/37 (35.1)
Median duration of sweats/night sweats (days) (*n* = 4)	29 (2–42)
Fatigue/malaise/weakness/asthenia/adynamic	15/40 (37.5)
Median duration of fatigue/malaise/weakness/asthenia/adynamic (days) (*n* = 8)	30 (14–365)
Hemoptysis	5/40 (12.5)

* Chronic cough is defined as cough of more than 8 weeks duration.

**Table 5:** Comparison of imaging presentation pre and post 1998.

	No/Total (%)	
	CXR	Chest CT
Before 1998		
Cavity	15/21 (71.4)	No chest CT conducted before 1998
Infiltrate	12/21 (57.1)	
Nodule	1/21 (4.8)	
Consolidation	2/21 (9.5)	
Fibrotic	0/21 (0)	
After 1998		
Cavity	4/18 (22.2)	2/11 (18.2)
Infiltrate	2/18 (11.1)	0/11 (0)
Nodule	9/18 (50)	9/11 (81.8)
Consolidation	3/18 (16.7)	0/11 (0)
Fibrotic	3/18 (16.7)	1/11 (9.1)

CXR—chest X-Ray; CT—computed tomography.

**Table 6:** Investigation, positive tests.

Investigation	Positive/Total Tests Conducted (%) (*n* = 39)
Culture	27/35 (77.1)
Antibody	21/27 (77.8)
Complement fixation test	16/20
Immunodiffusion	5/20
Histopathology	15/18 (83.3)
Histoplasmin skin test	11/18 (61.1)
Tuberculin skin test	7/16 (43.8)
Sputum smear microscopy for Histoplasma *	7/21 (33.3)
Antigen	1/3 (33.3)
PCR/NGS	1/4 (25)

* Only one publication mentioned the finding of intracellular yeast; PCR—polymerase chain reaction; NGS—next-generation sequencing.

**Table 7:** Culture positive samples.

Sample	No/Total
Sputum	19/27
Lung tissue	4/27
BAL	3/27
Mice inoculated intraperitoneally	1/27
Unspecified	1/27

Note: one culture was positive from sputum and lung tissue, and one culture from sputum and bronchoalveolar lavage (BAL).

**Table 8:** Investigation based on the case definition of pulmonary histoplasmosis.

Investigation	No/Total (%) Positive/Total
Proven (histopathology/culture/PCR)	36/39 (92.3)
Antibody (*n* = 24)	18/24
Antigen (*n* = 3)	1/3
Probable (antigen testing)	0/39
Possible (antibody testing)	3/39 (7.7)

PCR—polymerase chain reaction.

## Data Availability

No new data were created or analyzed in this study. Data sharing is not applicable to this article.

## Supplementary Materials

The following supporting information can be downloaded at: https://www.mdpi.com/article/10.3390/jof12030190/s1, Table S1: Studies excluded during the screening process and reasons for exclusion. Table S2: PRISMA 2020 Checklist: Locations of reporting items within the manuscript. Reference [64] is cited in the supplementary materials.
