# Supplementary material for: Misdiagnosis and Coinfection of Localized Pulmonary Histoplasmosis with Pulmonary Tuberculosis: A Systematic Review of Published Cases
Source: J Fungi (Basel). 2026 Mar 6;12(3):190. doi: 10.3390/jof12030190 (PMC13027406; doi:10.3390/jof12030190)
Supplement: Supplementary file 1 [file jof-12-00190-s001.zip › Supplementary Table S1. Studies excluded during the screening process and reasons for exclusion.pdf]

No.	Author(s) and Year	Study Title	Reason for Exclusion
1	Cronk et al. 1951	Pulmonary calcification and histoplasmin sensitivity in New York State.	Population not meeting diagnostic criteria
2	Schaefer et al. 1966	Pulmonary miliary calcification and histoplasmin sensitivity in Canadian eskimos.	Population not meeting diagnostic criteria
3	Furcolow et al. 1950	Tuberculin negative, histoplasmin positive, disseminated pulmonary calcification.	Population not meeting diagnostic criteria
4	Douglas et al. 1971	The dilemma of the asymptomatic pulmonary nodule in the young and not-so-young adult. Clinical conference in pulmonary disease from Northwestern University–McGaw Medical Center, Chicago.	Population not meeting diagnostic criteria
5	Agarwal et al. 2021	Acute pulmonary histoplasmosis masquerading as miliary tuberculosis in a non-endemic region.	Population not meeting diagnostic criteria
6	Soeroso et al. 2025	Chronic pulmonary histoplasmosis masquerading as tuberculoma: A case report and literature review.	Population not meeting diagnostic criteria
7	Venkataramana et al. 2016	Chronic Pulmonary Histoplasmosis and its Clinical Significance: an Under-reported Systemic Fungal Disease	Population not meeting diagnostic criteria
8	Denning et al. 2025	Radiological features of chronic pulmonary histoplasmosis: Easily mistaken for tuberculosis.	Review article
9	Garrido et al. 2006	Pulmonary histoplasmosis: unusual histopathologic findings.	Disseminated histoplasmosis
10	Jha et al. 2025	Pulmonary histoplasmosis with histoplasmosis tracheitis in a patient with systemic sclerosis: A case report and review of literature.	Disseminated histoplasmosis
11	Gianarakis et al. 2024	Granulomas Galore: Concomitant Granulomatous Infections in a Patient With Crohn's Disease.	Disseminated histoplasmosis

12	Bourne-Watrin et al. 2023	Pulmonary Histoplasmosis in People Living with Human Immunodeficiency Virus in French Guiana: Clinical Epidemiology, Medical Imaging and Prognostic.	Disseminated histoplasmosis
13	North et al. 1969	Bronchial and intercostal arteriography in non-neoplastic pulmonary disease.	Not TB related
14	Staszak et al. 2019	Mediastinal Lymphadenopathy in Children With Histoplasmosis.	Not TB related
15	Soni et al. 2025	Histoplasmosis in India: clinical insights from a tertiary care hospital.	Not TB related
16	Zöllner et al. 2010	Clinical and evolutionary characteristics of four patients with pulmonary histoplasmosis reported in the Paraíba Paulista Valley region.	Not TB related
17	dos Santos et al. 2004	Non-infectious and unusual infectious mimics of community-acquired pneumonia.	Not a misdiagnosis or coinfection between TB and histoplasmosis
18	Straub et al. 1962	Histoplasmosis, coccidioidomycosis and tuberculosis: a comparative pathological study.	Not a misdiagnosis or coinfection between TB and histoplasmosis
19	Oladele et al. 2022	revalence of Histoplasmosis among Persons with Advanced HIV Disease, Nigeria.	Not a misdiagnosis or coinfection between TB and histoplasmosis
20	Albandak et al. 2023	Chronic Cavitary Pulmonary Histoplasmosis in an Immunocompetent Patient.	Not a misdiagnosis or coinfection between TB and histoplasmosis
21	Larkin et al. 1964	Pulmonary histoplasmosis.	Not mention the clinic-radiologic profile
22	Chang et al. 2020	Granulomatous Inflammation Presenting as a Pulmonary Artery Mass.	Not mention the clinic-radiologic profile
23	Broaddus et al. 1985	Bronchoalveolar lavage and transbronchial biopsy for the diagnosis of pulmonary infections in the acquired immunodeficiency syndrome.	Not mention the clinic-radiologic profile
24	Huber et al. 2008	AIDS-related Histoplasma capsulatum var. capsulatum infection: 25 years experience of French Guiana.	Not mention the clinic-radiologic profile
25	Ekeng et al. 2022	Prevalence of Histoplasmosis and Molecular	Not mention the clinic-

		Characterization of Histoplasma species in Patients with Presumptive Pulmonary Tuberculosis in Calabar, Nigeria.	radiologic profile
26	Kusmiati et al. 2023	The seroprevalence of anti-Histoplasma capsulatum IgG antibody among pulmonary tuberculosis patients in seven referral tuberculosis hospitals in Indonesia.	Not mention the clinic-radiologic profile
27	Matsuda et al. 2021	Prevalence of pulmonary mycoses in smear-negative patients with suspected tuberculosis in the Brazilian Amazon.	Not mention the clinic-radiologic profile
28	Wijaya et al. 2024	Histoplasma antigen detection in unconfirmed pulmonary tuberculosis and cross-reactivity with Aspergillus antigen in patients and in food in Jakarta, Indonesia.	Not mention the clinic-radiologic profile
29	Soeroso et al. 2024	The Association of Chronic Pulmonary Aspergillosis and Chronic Pulmonary Histoplasmosis with MDR-TB Patients in Indonesia.	Not mention the clinic-radiologic profile
30	Dewi et al. 2023	Antibodies against Histoplasma capsulatum and Aspergillus fumigatus among chronic TB patients in Indonesia: a cross-sectional study.	Not mention the clinic-radiologic profile
31	Potosí et al. 2023	The relevance of clinical and epidemiological correlation in the early diagnosis of histoplasmosis: report of two clinical cases in Popayán, Colombia.	Article other than English language
