# Supplementary material for: Misdiagnosis and Coinfection of Localized Pulmonary Histoplasmosis with Pulmonary Tuberculosis: A Systematic Review of Published Cases
Source: J Fungi (Basel). 2026 Mar 6;12(3):190. doi: 10.3390/jof12030190 (PMC13027406; doi:10.3390/jof12030190)
Supplement: Supplementary file 1 [file jof-12-00190-s001.zip › Supplementary Table S2. PRISMA 2020 Checklist Locations of reporting items within the manuscript.pdf]

Section and Topic	Item #	Checklist Item	Location where item is reported
**TITLE**			
Title	1	Identify the report as a systematic review.	Title Page: The title includes "...and systematic review."
**ABSTRACT**			
Abstract	2	See the PRISMA 2020 for Abstracts checklist.	Abstract: The abstract describes the PRISMA 2020 protocol.
**INTRODUCTION**			
Rationale	3	Describe the rationale for the review in the context of existing knowledge.	Introduction, final paragraph: Describes the gaps and the need for this review.
Objectives	4	Provide an explicit statement of the objective(s) or question(s) the review addresses.	Introduction, final paragraph: "The aim of this review is to analyze…"
**METHODS**			
Eligibility criteria	5	Specify the inclusion and exclusion criteria for the review and how studies were grouped for the syntheses.	Section 2.2. ("Study eligibility criteria"): Describes the inclusion/exclusion criteria.
Information sources	6	Specify all databases, registers, websites, organisations, reference lists and other sources searched or consulted to identify studies. Specify the date when each source was last searched or consulted.	Section 2.1. ("Study design and setting"), Table 1: Search strategy and number of records
Search strategy	7	Present the full search strategies for all databases, registers and websites, including any filters and limits used.	Table 1: Complete search strings and records found on PubMed, EBSCOhost, ProQuest, BioRxiv,

			MedRxiv
**Section and Topic**	**Item #**	**Checklist Item**	**Location where item is reported**
Selection process	8	Specify the methods used to decide whether a study met the inclusion criteria of the review, including how many reviewers screened each record and each report retrieved, whether they worked independently, and if applicable, details of automation tools used in the process.	Section 2.3. ("Data screening and selection"): Describes the screening and evaluation process. Figure 1(PRISMA Flowchart): Illustrates the process.
Data collection process	9	Specify the methods used to collect data from reports, including how many reviewers collected data from each report, whether they worked independently, any processes for obtaining or confirming data from study investigators, and if applicable, details of automation tools used in the process.	Section 2.3. ("Data screening and selection"): Describes the data collection method, Table 2: Evaluation of studies using the Joanna Briggs Institute's (JBI) appraisal criteria
Data items	10a	List and define all outcomes for which data were sought. Specify whether all results that were compatible with each outcome domain in each study were sought (e.g. for all measures, time points, analyses), and if not, the methods used to decide which results to collect.	Section 2.4. ("Data extraction"): Describes data extracted from each eligible study
	10b	List and define all other variables for which data were sought (e.g. participant and intervention characteristics, funding sources). Describe any assumptions made about any missing or unclear information.	When information was missing or unclear, it was recorded as reported without assumptions.
Study risk of bias assessment	11	Specify the methods used to assess risk of bias in the included studies, including details of the tool(s) used, how many reviewers assessed each study and whether they worked independently, and if applicable, details of automation tools used in the process.	Risk of bias was assessed using the Joanna Briggs Institute's (JBI) appraisal criteria, Table 2

Section and Topic	Item #	Checklist Item	Location where item is reported
Effect measures	12	Specify for each outcome the effect measure(s) (e.g. risk ratio, mean difference) used in the synthesis or presentation of results.	N/A. It is not a review of clinical or intervention trials.
Synthesis methods	13a	Describe the processes used to decide which studies were eligible for each synthesis (e.g.tabulating the study intervention characteristics and comparing against the planned groups for each synthesis (item #5)).	Section 2.4. and Section 2.5. ("Data synthesis"): Describes how studies were grouped, that is according to the topics defined in the review (demographic and baseline characteristics, clinical manifestation, imaging results, laboratory findings, and investigation methods)
	13b	Describe any methods required to prepare the data for presentation or synthesis, such as handling of missing summary statistics, or data conversions.	Section 2.5.: Describes data organization and presentation. No data conversions or imputations were needed. Missing information was kept as reported in the original studies.
	13c	Describe any methods used to tabulate or visually display results of individual studies and syntheses.	Results were summarized in tables and figures to display patterns across studies. Tables 3-8
	13d	Describe any methods used to synthesize results and provide a rationale for the choice(s). If meta-analysis was performed, describe the model(s), method(s) to identify the presence and extent of statistical heterogeneity, and software package(s) used.	The synthesis is narrative, organizing the findings of the 24 studies into coherent themes. A narrative synthesis was performed. Findings from the included studies were organized into major themes to summarize trends, methodological differences, and reported outcomes. No meta-

			analysis was conducted
	13e	Describe any methods used to explore possible causes of heterogeneity among study results (e.g. subgroup analysis, meta-regression).	N/A. No quantitative meta-analysis was performed.
	13f	Describe any sensitivity analyses conducted to assess robustness of the synthesized results.	N/A. Sensitivity analysis does not apply to narrative syntheses.
Reporting bias assessment	14	Describe any methods used to assess risk of bias due to missing results in a synthesis (arising from reporting biases).	Only available data were recorded, missing results were excluded
Certainty assessment	15	Describe any methods used to assess certainty (or confidence) in the body of evidence for an outcome.	Table 2: Joanna Briggs Institute's (JBI) appraisal criteria was used to evaluate the 24 studies for completeness of data.
**RESULTS**			
Study selection	16a	Describe the results of the search and selection process, from the number of records identified in the search to the number of studies included in the review, ideally using a flow diagram.	Table 1: Search strategy and number of records, and Figure 1.
	16b	Cite studies that might appear to meet the inclusion criteria, but which were excluded, and explain why they were excluded.	The details on excluded study were shown on supplementary material 2.
Study characteristics	17	Cite each included study and present its characteristics.	The included studies are cited and discussed throughout the results section and listed in the references. Table 3 describes demographic and baseline characteristics specified to each study.
Risk of bias in	18	Present assessments of risk of bias for	Risk of bias was assessed

studies		each included study.	using the Joanna Briggs Institute's (JBI) appraisal criteria, Table 2.
Results of individual studies	19	For all outcomes, present, for each study: (a) summary statistics for each group (where appropriate) and (b) an effect estimate and its precision (e.g. confidence/credible interval), ideally using structured tables or plots.	Tables 3-8 presents summary statistics for each outcome category (demographic and baseline characteristics, clinical manifestation, imaging results, laboratory findings, and investigation methods).
	20a	For each synthesis, briefly summarise the characteristics and risk of bias among contributing studies.	The characteristics and risk of bias among contributing studies is summarized in Table 2.
	20b	Present results of all statistical syntheses conducted. If meta-analysis was done, present for each the summary estimate and its precision (e.g. confidence/credible interval) and measures of statistical heterogeneity. If comparing groups, describe the direction of the effect.	N/A. There was no statistical synthesis.
	20c	Present results of all investigations of possible causes of heterogeneity among study results.	N/A.
	20d	Present results of all sensitivity analyses conducted to assess the robustness of the synthesized results.	N/A.
Reporting biases	21	Present assessments of risk of bias due to missing results (arising from reporting biases) for each synthesis assessed.	Only available data were recorded, missing results were excluded
Certainty of evidence	22	Present assessments of certainty (or confidence) in the body of evidence for each outcome assessed.	Table 2: Joanna Briggs Institute's (JBI) appraisal criteria was used to evaluate the 24 studies for completeness of data.

DISCUSSION			
Discussion	23a	Provide a general interpretation of the results in the context of other evidence.	This review confirms prior evidence that pulmonary histoplasmosis closely mimics tuberculosis clinically and radiologically, leading to frequent misdiagnosis or coinfection. Reported risk factors, symptoms, and imaging findings are consistent with previous studies from endemic regions, reinforcing known epidemiologic and diagnostic challenges.
	23b	Discuss any limitations of the evidence included in the review.	The evidence is limited by reliance on heterogeneous case reports and case series, small sample sizes, incomplete diagnostic testing, and underrepresentation of regions such as Southeast Asia. Older cases and diagnostic variability precluded quantitative synthesis and may not reflect current epidemiology.
	23c	Discuss any limitations of the review processes used.	This review included only English-language publications, introducing potential language and publication bias, as well as underrepresentation of regions especially in Asia.
	23d	Discuss implications of the results for practice, policy, and future research.	Clinicians in TB-endemic settings should consider histoplasmosis when TB tests are negative or treatment fails. Improved

			access to serology, antigen testing, and CT imaging is needed. Future research should include multicenter studies, non-English literature, and standardized diagnostic approaches.
**OTHER INFORMATION**			
Registration and protocol	24a	Provide registration information for the review, including register name and registration number, or state that the review was not registered.	Section 2.2: "The protocol of this systematic review was registered to open science framework (OSF) registries".
	24b	Indicate where the review protocol can be accessed, or state that a protocol was not prepared.	The protocol of this systematic review can be accessed through the link https://doi.org/10.17605/OSF.IO/ES9YH". (Section 2.2)
	24c	Describe and explain any amendments to information provided at registration or in the protocol.	N/A
Support	25	Describe sources of financial or non-financial support for the review, and the role of the funders or sponsors in the review.	Section "Funding": "This research received no external funding".
Competing interests	26	Declare any competing interests of review authors.	Section "Conflicts of Interest": "All the authors declare that there are no conflicts of interest."
Availability of data, code and other materials	27	Report which of the following are publicly available and where they can be found: template data collection forms; data extracted from included studies; data used for all analyses; analytic code; any other materials used in the review.	Section "Data Availability Statement": "Upon reasonable request, data can be shared by contacting the corresponding author".

From: Page MJ, McKenzie JE, Bossuyt PM, Boutron I, Hoffmann TC, Mulrow CD, et al. The PRISMA 2020 statement: an updated guideline for reporting systematic reviews. BMJ 2021;372: n71. doi: 10.1136/bmj.n71. This work is licensed under CC BY 4.0. To view a copy of this license, visit https://creativecommons.org/licenses/by/4.0/
